# A study of the relationship between social support, depression, alexithymia and glycemic control in patients with type 2 diabetes mellitus: a structural equation modeling approach

**DOI:** 10.3389/fendo.2024.1390564

**Published:** 2024-08-20

**Authors:** Yuqin Gan, Fengxiang Tian, Xinxin Fan, Hui Wang, Jian Zhou, Naihui Yang, Hong Qi

**Affiliations:** ^1^ Clinical Medical College of Chengdu Medical College, First Affiliated Hospital, Chengdu, China; ^2^ The Fourth Hospital of West China, Sichuan University, Chengdu, China; ^3^ School of Nursing, Chengdu Medical College, Chengdu, China; ^4^ Nursing Department, First Affiliated Hospital of Chengdu Medical College, Chengdu, China; ^5^ Department of Rheumatology and Immunology, The First Affiliated Hospital of Chengdu Medical College, Chengdu, China

**Keywords:** type 2 diabetes mellitus, glycemic control, alexithymia, social support, depression, structural equation modeling

## Abstract

**Aim:**

The aim of this research was to ascertain the correlations between alexithymia, social support, depression, and glycemic control in patients diagnosed with type 2 diabetes mellitus. Additionally, this study sought to delve into the potential mediating effects of social support and depression in the relationship between alexithymia and glycemic control.

**Method:**

A purposive sampling methodology was employed to select a cohort of 318 patients afflicted with type 2 diabetes mellitus, hailing from a care establishment situated in Chengdu City. This investigation embraced a cross-sectional framework, wherein instruments such as the General Information Questionnaire, the Toronto Alexithymia Scale 20, the Social Support Rating Scale, and the Hamilton Depression Scale were judiciously administered. The primary objective of this endeavor was to unravel the interplay that exists amongst alexithymia, social support, depression, and glycemic control. The inquiry discerned these interrelationships through both univariate and correlational analyses, subsequently delving into a comprehensive exploration of the mediating ramifications engendered by social support and depression in the nexus between alexithymia and glycemic control.

**Results:**

The HbA_1c_ level of patients diagnosed with type 2 diabetes mellitus was recorded as (8.85 ± 2.107), and their current status with regards to alexithymia, social support, and depression were measured as (58.05 ± 4.382), (34.29 ± 4.420), and (7.17 ± 3.367), respectively. Significant correlations were found between HbA_1c_ and alexithymia (R=0.392, *P*<0.01), social support (R=-0.338, *P*<0.01), and depression (R=0.509, *P*<0.01). Moreover, alexithymia correlation with social support (R=-0.357, *P*<0.01) and with depression (R=0.345, *P*<0.01). Regarding the mediation analysis, the direct effect of alexithymia on HbA_1c_ was calculated to be 0.158, while the indirect effect through social support and depression were 0.086 and 0.149, respectively. The total effect value was determined to be 0.382, with the mediating effect accounting for 59.95%, and the direct effect accounting for 40.31%.

**Conclusion:**

Alexithymia exerts both direct and indirect adverse effects on glycemic control, thereby exacerbating disease outcomes. Hence, it is imperative to prioritize the mental health status of individuals with type 2 diabetes to enhance overall well-being, ameliorate diabetes-related outcomes, elevate patients’ quality of life, and alleviate the psychological distress and financial burden associated with the condition.

## 1 Introduction

In accordance with a survey conducted by the International Diabetes Federation(IDF) (1), the global prevalence of diabetes mellitus is anticipated to reach approximately 10.5% (536.6 million) in 2021, projecting an escalation to 12.2% (783.2 million) by the year 2045. In tandem with the rapid advancement of China’s economy and urbanization, coupled with elevated living standards and population aging, among other factors, the annual increment in the prevalence of diabetes mellitus has become discernible (2). The prevalence of diabetes mellitus among individuals aged 18 years and above is presently documented at 11.2%, representing the highest prevalence nationwide, with type 2 diabetes mellitus (T2DM) constituting over 90% of these cases (3). Enhancing the degree of glycemic control to impede the progression of complications stands as the principal therapeutic objective for individuals with diabetes (4). It serves as the quintessential standard for averting both microvascular and macrovascular complications in the context of diabetes mellitus (5). Clinically, glycated hemoglobin (HbA_1c_) emerges as the prevailing benchmark for assessing glycemic control, with a diagnostic threshold set at 7.0% (6). Maintaining blood glucose concentrations within the normative range has the potential to diminish both the frequency and severity of diabetic complications. Conversely, an elevated HbA_1c_ level signifies suboptimal glycemic control over the preceding 2-3 months, thereby escalating the susceptibility to complications, encompassing both microvascular and macrovascular manifestations (7). Prolonged hyperglycemia not only heightens the risk of such complications but also amplifies the likelihood of mortality among affected individuals. Notably, the glycemic control rate among Chinese diabetic patients stands at a modest 50.1% (6), indicating a subpar level that warrants further enhancement. Henceforth, it becomes imperative to ameliorate the degree of glycemic control among individuals afflicted with T2DM with the dual objective of impeding the progression of diabetic complications and concurrently mitigating the psychological burden borne by the patients. Such interventions aim to actualize the enhancement of patients’ quality of life and the alleviation of the associated economic burdens.

T2DM exerts a substantial financial encumbrance upon individuals and their families, owing to its irreversible nature, protracted duration, recurring nature, myriad complications, and the elevated costs associated with its treatment. Liu et al. (8)prognosticate that the aggregate expenditure for adult diabetes in China will surge from US$250.2 billion in 2020 to US$460.4 billion in 2030. This escalation, reflecting an annual growth rate of 6.32% over the period from 2020 to 2030 (5.99% - 6.65%), surpasses the rate of Gross Domestic Product (GDP) growth. This financial burden encompasses both direct costs linked to the prevention and treatment of diabetes and its associated complications, as well as indirect costs encompassing disability, loss of work productivity, and mortality (9). Moreover, the enduring nature of diabetes treatment elevates the susceptibility of patients to psychological disorders (10). These psychological disturbances, in turn, precipitate diminished adherence to treatment regimens and self-management protocols, thereby fostering suboptimal glycemic control. Consequently, the escalated risk of diabetes-related complications and mortality ensues, culminating in a deterioration of patients’ quality of life and an augmentation of healthcare expenditures (11). The involvement of psychological factors in the etiopathogenesis of chronic diseases is awakening the interest of the scientific community (12). Empirical investigations (13) delineate that the incidence of psychological disorders among diabetic patients surpasses that of their non-diabetic counterparts by more than twofold. Consequently, there exists an exigency to enhance the mental well-being of diabetic patients with a view to ameliorating their quality of life and alleviating the associated economic burdens. The prevalence of alexithymia among diabetic patients exceeds that observed in the general population (14). Notably, its detection rate reaches as high as 75.8% in diabetic patients from foreign cohorts (15), and up to 45% among older people diabetic individuals in China, reflecting an upward trajectory (16, 17). Alexithymia exerts adverse effects on clinical manifestations, disease perception, severity, progression, and treatment adherence. These repercussions, in turn, contribute to unfavorable disease outcomes, a diminished quality of life for patients (18, 19), and an augmented risk of mortality (20). Fares et al. (21) discerned a positive correlation between alexithymia and glycemic control in patients diagnosed with T2DM. Notably, the incidence of severe hyperglycemic episodes was threefold higher among individuals with alexithymia compared to those devoid of this psychological disposition. Furthermore, hospitalizations due to hyperglycemia were five times more frequent in patients exhibiting alexithymia compared to their counterparts lacking this psychological trait.

Social support assumes a buffering role in mental health, serving as a protective mechanism against the onset of physical and psychological disorders induced by heightened stress (22). It constitutes a pivotal element in fostering the treatment efficacy and recuperative processes in individuals diagnosed with diabetes (23). Furthermore, social support facilitates enhanced self-management strategies, thereby ameliorating patients’ lifestyles and fortifying disease management, ultimately contributing to an augmentation in glycemic control (24). Its affirmative impact is conspicuous in the context of disease treatment and recuperation (23). Diminished levels of social support can amplify the incidence of alexithymia by constricting the patient’s social milieu, inducing feelings of isolation, and curtailing the capacity to engage in dialogue or express emotions during periods of heightened psychological stress (25).

Concurrently, the progression of diabetes mellitus, coupled with the protracted course of treatment, precipitates the development of complications and an escalation in treatment expenses. Consequently, there is a commensurate augmentation in the psychological burden borne by the patient, with depression emerging as one of the most prevalent negative emotional outcomes (26). The prevalence of depression in individuals with diabetes ranges from 22% to 62% and, in some instances, may ascend to 73% (27), reflecting a prevalence approximately fivefold higher than that observed in the general population (28). Depression is associated with an elevated incidence of complications in diabetic patients, contributing to an increased disability rate and a curtailed life expectancy (29, 30). Moreover, it amplifies mortality rates by approximately 110% (31). Depression further engenders the manifestation of severe psychological symptoms in individuals with T2DM, fostering diminished treatment adherence, exerting a discernible impact on glycemic control, and augmenting the prevalence of alexithymia (32, 33).

In conclusion, a correlation exists among social support, depression, alexithymia, and glycemic control in patients diagnosed with T2DM; however, no study has systematically investigated the precise mechanistic pathways interconnecting these four variables. The current study delved into elucidating the roles of social support, depression, and alexithymia in influencing glycemic control, thereby establishing a foundational framework for clinical practitioners to enhance glycemic control strategies for individuals with T2DM.

## 2 Materials and methods

### 2.1 Study design and participants

This study is of a cross-sectional nature, and it recruited individuals diagnosed with T2DM who sought medical care within the endocrine inpatient department and outpatient clinic of a tertiary healthcare facility situated in Chengdu during the period spanning from October 2022 to June 2023.Inclusion criteria: ① Patients who conformed to the diagnostic standards set forth by the World Health Organization in 1999 for T2DM (34). ② Patients with a confirmed T2DM diagnosis for a duration of no less than 6 months. ③Age range: 18 years to 80 years. ④Cognitively sound, capable of regular communication, and possessing a comprehensive understanding of the questionnaire’s content. ⑤Individuals who have provided informed consent and willingly enrolled in this investigation. Exclusion criteria: ① Patients presenting severe chronic ailments, such as those affecting the cardiovascular, cerebral, hepatic, renal, or pulmonary systems; ② Patients afflicted with psychiatric disorders or cognitive impairments (excluding depressed patients); ③ Patients in critical medical states, precluding their ability to collaborate with the investigative procedures.

Sample size calculation:In accordance with the Kendall sample size estimation method, the sample size was determined to be a minimum of ten times the number of variables (35). This study incorporated four research instruments, which included a 12-item General Information Questionnaire, a 3-item Social Support Rating Scale, and a 5-item Hamilton Depression Scale, a 3-item Toronto Narrative Alexithymia Scale, totaling 23 items. Hence, the total sample size comprised 230 cases. To safeguard against potential sample attrition influencing the study outcomes, a 20% sample loss margin was incorporated, resulting in a final sample size of 276 cases, as dictated by the requirements of structural equation modeling. The final sample size of 318 cases was included in conjunction with the actual clinical survey

### 2.2 Data collection

Prior to commencing the survey, the researcher (XF) engaged in a comprehensive review of the questionnaire’s content. Additionally, any queries or uncertainties were addressed through consultation with pertinent experts or professionals. Throughout the survey process, the researcher elucidated the study’s protocol to the participating patients. Those who consented to participate formally by signing the written informed consent document were subsequently entrusted to independently complete the questionnaires following standardized instructions provided by the researcher. In instances where participants encountered difficulties during the questionnaire completion, the researcher offered appropriate assistance. Upon the conclusion of the questionnaire administration, the researcher collected the completed forms on-site to ensure their comprehensive fulfillment and promptly addressed any vacancies requiring supplementation.

### 2.3 Ethics approval

The study was approved by the Ethics Committee of the First Affiliated Hospital of Chengdu Medical College (2022CYFYIRB-BA-Oct19), and the subjects signed an informed consent form before the investigation.

### 2.4 Research instruments

#### 2.4.1 General information questionnaire

12 entries, including gender, age, education, marital status, occupation, per capita monthly household income, presence of health insurance, duration of illness, treatment modalities, presence of complications, co-morbidities, HAb_1c_.

#### 2.4.2 The social support rating scale

Devised by Chinese psychologist Xiao Shuiyuan (36) in 1986 for the assessment of individual social support, exhibits commendable psychometric properties. The scale contains three dimensions of objective support (3 entries), subjective support (4 entries), and utilization of social support (3 entries), for a total of 10 entries, demonstrates a high level of internal consistency with Cronbach’s alpha coefficients ranging from 0.89 to 0.94 for both the overall scale and its constituent dimensions, alongside an impressive retest reliability of 0.92. Huang Zizin et al. (25) applied this scale to patients with T2DM, revealing a slightly reduced but still acceptable Cronbach’s alpha coefficient of 0.72 for the overall scale. In the present study, the Cronbach’s alpha coefficients for both the total scale and its dimensions ranged from 0.828 to 0.952, reaffirming its reliability. Interpretation of the questionnaire scores is as follows: Scores between 12 and 22 are indicative of a low level of social support, scores ranging from 23 to 43 denote a moderate level of social support, and scores falling within the range of 44 to 66 signify a high level of social support.

#### 2.4.3 The Hamilton depression scale

Employed to assess the degree of patients’ depressive condition, comprises 17 items distributed with 17 entries and 5 factors, i.e., somatization of anxiety, weight, cognitive impairment, silted up, and sleep disturbance. As originally reported by Hamilton himself, the scale exhibits a Cronbach’s alpha coefficients of 0.90, while foreign studies attest to a validity exceeding 0.84. The reliability of the 1988 Chinese version of this scale demonstrates excellence, with empirical veracity coefficients within domestic literature reflecting a substantial clinical symptom severity coefficient of 0.92. Interpretation of the questionnaire’s total score is as follows: Scores falling within the range of ≤ 7 points are indicative of a normal state, while scores ranging from 8 to 17 points denote mild depression, scores of 18 to 24 points represent moderate depression, and scores equal to or exceeding 25 points signify severe depression.

#### 2.4.4 Toronto alexithymia scale 20

The TAS-20, developed by Taylor et al. (37) in 1984 and subsequently adapted by Bagby et al. (38) to create the Toronto Alexithymia Scale TAS-26, underwent translation and revision to yield the Chinese version by Yao Shuqiao et al. (39). The scale exhibits commendable psychometric properties, boasting a Cronbach’s alpha coefficient of 0.83 and retest reliability of 0.87. The scale consists of 20 entries with 3 factors: identifying affective disorders (7 entries), describing affective disorders (5 entries) and extraverted thinking (8 entries). The TAS-20 serves as a universally applicable and widely employed tool for assessing alexithymia, characterized by robust reliability and validity. A questionnaire score equal to or below 60 signifies the absence of alexithymia, whereas a score equal to or exceeding 61 indicates the presence of alexithymia.

### 2.5 Statistical analysis

The data were exported from the EpiData management software (Chinese version) and subjected to analysis using IBM SPSS 26.0 software. Quantitative data were presented as mean ± standard deviation (`x ± s), while qualitative data were expressed in terms of case count and percentage (%). Linear regression was employed to scrutinize the impact of social support, depression, and alexithymia on glycemic control, and Pearson’s correlation was utilized to investigate the interrelations among these variables. The construction of a structural equation model for factors impacting glycemic control in patients with type 2 diabetes mellitus was executed using AMOS 26.0 software. This encompassed an evaluation based on several goodness-of-fit indices, namely Goodness of Fit Index (GFI), Incremental Fit Index (IFI), Comparative Fit Index (CFI), Standardized Fit Index (NFl), Relative Fit Index (RFI), Non-normalized Fit Index (TLI), Normed Fit Index (NFl), and Root Mean Square Error of Approximation. The indices IFI, CFI, NFI, RFI, and TLI all exceeded 0.9, with RMSEA below 0.08, and 2/DF below 3, adhering to accepted standards (40). A statistically significant difference was ascribed to instances with a P-value less than 0.05.

## 3 Results

### 3.1 Participant characteristics

A total of 318 study participants were enrolled in this investigation, comprising 152 males (47.8%) and 166 females (52.2%). Their age distribution was as follows: 22 individuals (6.9%) aged 18-44, 174 individuals (54.7%) aged 45-64, and 122 individuals (38.4%) aged 65 and above. In terms of marital status, 267 participants (84.0%) were married, while 51 participants (16.0%) were not. Employment status revealed 98 participants (30.8%) were employed, and 220 participants (69.2%) were not actively working. Health insurance coverage was prevalent, with 295 participants (92.8%) having it, while 23 participants (7.2%) did not possess health insurance. Additional demographic details of the study cohort are delineated in **Table 1**.

**Table 1:** Sociodemographic characteristics.

Characteristics	Category	n(%)
Gender	Man	152(47.8)
	Woman	166(52.2)
Age (years)	18-44	22(6.9)
	45-64	174(54.7)
	≥65	122(38.4)
Education level	Primary and below	132(41.5)
	middle school	114(35.8)
	high school or junior college	48(15.1)
	college and above	24(7.5)
Marital status	marriage	267(84.0)
	non-marital	51(16.0)
Career	incumbency	98(30.8)
	non-working	220(69.2)
Monthly per capita family income	<3000	139(43.7)
	3000-5000	103(32.4)
	>5000	76(23.9)
Medical insurance	Yes	295(92.8)
	No	23(7.2)
Course of disease(year)	1-10	203(63.8)
	11-20	88(27.7)
	≥21	27(8.5)
Treatment	dietary control only	24(7.5)
	antihyperglycemic drug	165(51.9)
	insulin	43(13.5)
	glucose-lowering drugs and insulin	86(27.0)
Complications	Yes	113(35.5)
	No	205(64.5)
Co-morbidity	Yes	166(52.2)
	No	152(47.8)
HbA_1c_	≤7.0	78(24.5)
	>7.0	240(75.5)

### 3.2 Current status of glycemic control, alexithymia, social support and depression in T2DM patients

The glycemic control level among T2DM patients was determined to be (8.85 ± 2.107), while the indices for alexithymia, social support, and depression were measured at (58.05 ± 4.382), (34.29 ± 4.420), and (7.17 ± 3.367), respectively. Notably, suboptimal glycemic control was evident in 75.47% of cases, with 31.13% of participants exhibiting alexithymia, and a significant 94.97% experiencing an intermediate level of social support. Furthermore, depressive symptoms were reported by 45.6% of the participants. Detailed findings are presented in **Table 2**.

**Table 2:** Glycemic control, alexithymia, social support and depression scores in T2DM patients (M ± SD).

	Current situation	M ± SD	Entry M ± SD
HAb_1c_	<7.0%(78,24.53%) ≥7.0%(240,75.47%)	8.85 ± 2.107	—
Alexithymia	No(219,68.87%) Yes(99,31.13%)	58.05 ± 4.382	—
DIF		18.31 ± 3.153	2.62 ± 0.450
DDF		14.39 ± 1.718	2.88 ± 0.344
EOT		25.35 ± 1.897	3.17 ± 0.237
Social support	Low level(1,0.31%) Medium level(302,94.97%) High level(15,4.72%)	34.29 ± 4.420	—
OS		10.02 ± 2.264	3.34 ± 0.755
SS		19.36 ± 2.684	4.84 ± 0.671
USS		4.92 ± 1.658	1.64 ± 0.553
Depression	No(173,54.40%) Mildly(140,44.03%) Moderately(5,1.57%)	7.17 ± 3.367	—
SA		2.02 ± 1.475	0.40 ± 0.295
W		1.22 ± 1.533	1.22 ± 1.533
CI		0.64 ± 0.788	0.21 ± 0.263
SU		0.82 ± 1.145	0.21 ± 0.286
SD		2.37 ± 1.493	0.79 ± 0.498

DIF, Difficulty Identifying Feelings; DDF, Difficulty Describing Feelings;

EOT, Externally-Oriented Thinking; OS, objective support; SS, subjective support; USS, utilization of social support; SA, Somatization of anxiety; W, weight; CI, cognitive impairment; SU, silted up; SD, sleep disorder.

### 3.3 Univariate analysis of factors influencing glycemic control in T2DM patients

A linear regression analysis concerning alterations in HbA_1c_ was performed, with HbA_1c_ serving as the dependent variable and alexithymia, social support, and depression acting as independent variables. The findings underscored that alexithymia, social support, and depression emerged as significant determinants influencing HbA_1c_ levels in patients diagnosed with T2DM (*P*<0.05). Elaborative outcomes are delineated in **Table 3**.

**Table 3:** Linear regression analysis of factors affecting HbA_1c_ in patients with type 2 diabetes mellitus.

Dependent variable	Independent variable	β	standardized coefficient β	t	P-Value	β95%CI	
						Lower	Upper
HbA_1c_	alexithymia	0.189	0.392	7.578	<0.001	0.140	0.237
	social support	-0.161	-0.338	-6.375	<0.001	-0.211	-0.111
	depression	0.318	0.509	10.510	<0.001	0.259	0.378

### 3.4 Analysis of the correlation between glycemic control, alexithymia, social support and depression in T2DM patients

Pearson correlation analysis was employed to scrutinize the associations among social support, depression, alexithymia, and blood glucose control in individuals diagnosed with T2DM. The outcomes revealed that HbA_1c_ exhibited a positive correlation with both alexithymia (r=0.392, *P*<0.01) and depression (r=0.509, *P*<0.01), while demonstrating a negative correlation with social support (r=-0.338, *P*<0.01). Furthermore, alexithymia displayed a negative correlation with social support (r=-0.357, *P*<0.01) and a positive correlation with depression (r=0.345, *P*<0.01). Notably, social support exhibited no significant association with depression (r=-0.095, *P*>0.05). Comprehensive details are available in **Table 4**.

**Table 4:** Correlations between social support, depression, alexithymia, and glycemic control in patients with type 2 diabetes mellitus.

	HbA_1c_	alexithymia	social support	depression
HbA_1c_	1			
alexithymia	0.392**	1		
social support	-0.338**	-0.357**	1	
depression	0.509**	0.345**	-0.095	1

**P<0.01.

### 3.5 Structural equation modeling between for the study of glycemic control in T2DM patients

**Figure 1:**
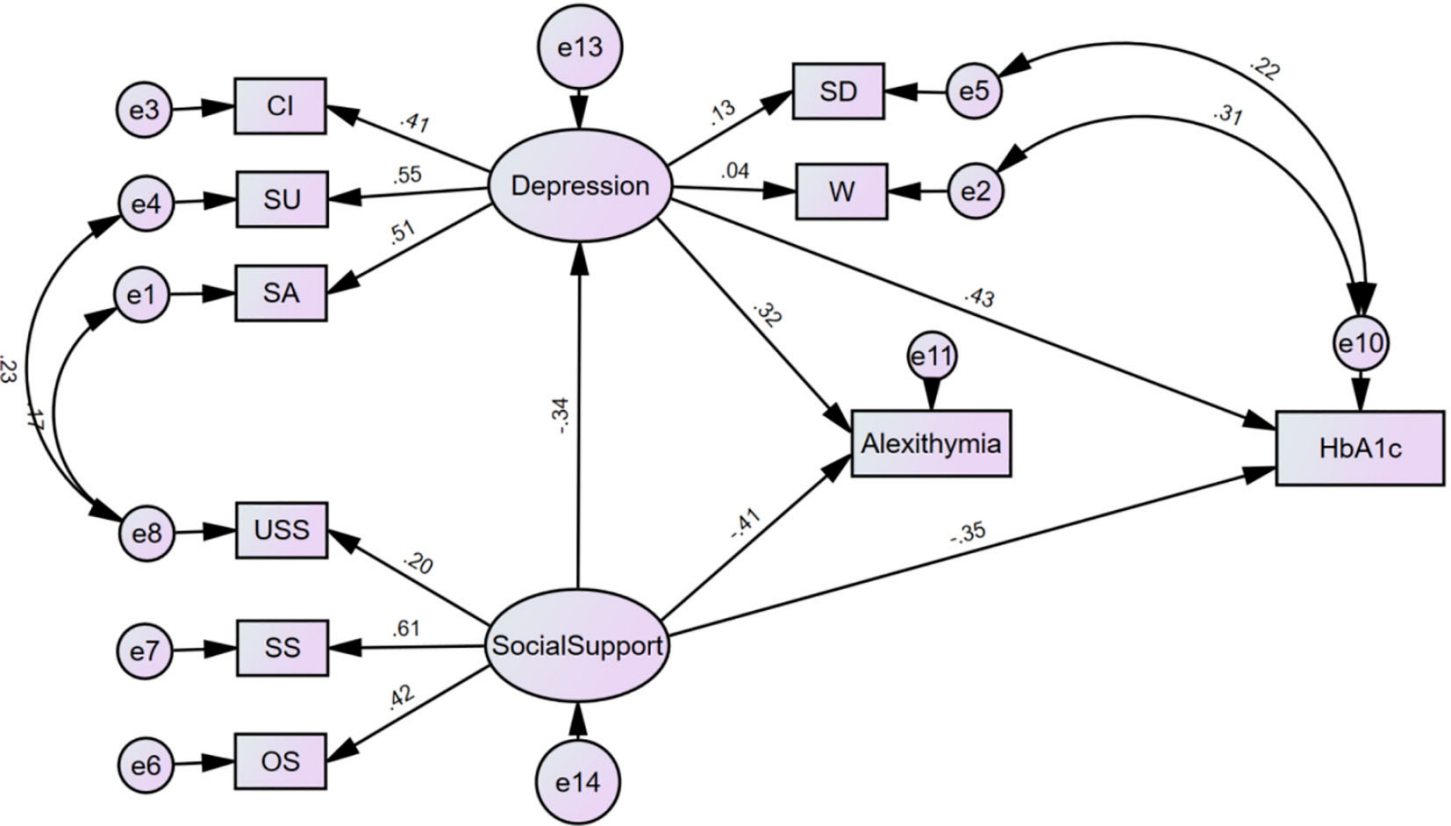
Modified model of glycemic control in patients with types 2 diabetes mellitus. USS, utilization of social support; SS, subjective support; OS, objective support; Cl, cognitive impairment; SU, silted up; SA, somatization of anxiety; SD, sleep disorder; W, weight.

Derived from the initial model outcomes, inoperative paths were excised, and the initial model underwent refinement through amalgamation with correction indices. This culminated in the formulation of the definitive structural equation model delineating glycemic control in individuals diagnosed with T2DM, as elucidated in **Figure 1**. The model, presented in a standardized format, encompasses standardized path coefficients. Subsequently, the revised model was re-fitted to the dataset employing the maximum likelihood method. The ensuing results indicated commendable fit indices, including RMSEA=0.043 (<0.08), χ2/df=2.577 (<3), GFI=0.973 (>0.9), AGFI=0.948 (>0.9), IFI=0.952 (>0.9), TLI=0.918 (>0.9), and CFI=0.949 (>0.9), all well within the normative range of values. Additionally, NFI=0.878 (<0.9) and RFI=0.804 (<0.9), though marginally below 0.9, still fall within the acceptable threshold, affirming the enhanced fit of the refined model. For comprehensive specifics, refer to **Table 5**.

**Table 5:** Evaluation results of the optimal model fit goodness-of-fit.

Adaptation index	χ^2^/df	GFI	NFI	RFI	IFI	TLI	CFI	RMSEA
Reference value	<3.00	>0.9	>0.9	>0.9	>0.9	>0.9	>0.9	<0.08
Model test value	1.577	0.973	0.878	0.804	0.952	0.918	0.949	0.043

### 3.6 Effect analysis of structural equation modeling variables

The refined model exhibited fitting indices within acceptable parameters. The model outcomes indicated that social support manifested a negative association with depression (β=-0.336, t=-2.398, *P*=0.016), and likewise, social support displayed a negative correlation with alexithymia (β=-0.405, t=-3.566, *P*<0.001). Moreover, social support revealed an inverse relationship with glycemic control (β=-0.346, t=-3.437, *P*<0.001). Conversely, depression exhibited a positive connection with alexithymia (β=0.318, t=3.233, *P*=0.001) and also demonstrated a positive correlation with glycemic control (β=0.434, t=4.168, *P*<0.001). Notably, alexithymia exhibited no statistically significant relationship with glycemic control (*P*>0.05), as elucidated in **Table 6**.

**Table 6:** Parameter estimation of a modified model of social support, depression, alexithymia and glycemic control in patients with type 2 diabetes mellitus.

	Standardized coefficient	S.E.	C.R.	*P*
depression<—social support	-0.336	0.112	-2.398	0.016
alexithymia<—social support	-0.405	0.530	-3.566	***
HAb_1c_<—social support	-0.346	0.225	-3.437	***
alexithymia<—depression	0.318	0.570	3.233	0.001
HAb_1c_<—depression	0.434	0.290	4.168	***

SE denotes standard error, C.R. denotes critical ratio, i.e., t-value, and *** is P < 0.001.

## 4 Discussion

The study findings indicated that social support exerts a direct and indirect impact on glycemic control through depression. Social support was observed to have a direct effect on alexithymia. Depression exhibited direct influences on both alexithymia and glycemic control. Furthermore, a correlation was established between alexithymia and glycemic control; however, the specific pathways connecting these two variables remain unconfirmed within the scope of this investigation. This study establishes a theoretical foundation for elucidating the impact of social support, depression, and alexithymia on glycemic control through an examination of the intricate pathways interconnecting social support, depression, and alexithymia with glycemic control. Furthermore, it furnishes theoretical substantiation for enhancing glycemic control in individuals with T2DM with the ultimate goal of ameliorating the overall glycemic control in T2DM patients. The overarching objective is to impede the progression of complications, thereby enhancing the quality of life for patients, while concurrently mitigating the perceptual and economic burdens associated with the disease.

### 4.1 Current status of social support in T2DM patients

The study results revealed that the comprehensive social support score for patients diagnosed with T2DM was (34.29 ± 4.42), indicating a moderate level. This finding aligns with the research conducted by Al-Dwaikat et al. (41) and contrasts with the outcomes reported by Qin Wen et al. (42) (39.27 ± 8.82), where social support levels were higher. Specifically, the subjective support score ranked highest, followed by the objective support score, while the social support utilization score was the lowest. This pattern resonates with the outcomes of a social support survey for diabetic patients conducted by Liu Qing et al. (43). Notably, patients exhibited a relatively high subjective perception of acquiring social support; however, the practical benefits derived from this assistance were diminished, impeding their ability to fully harness external aid. This suboptimal utilization of support resulted in consequences such as social isolation and delayed medical intervention (44), thereby influencing the efficacy of disease treatment. Social support not only exerts a direct positive influence on well-being but also functions as a buffer, shielding individuals from health issues induced by excessive stress (22). Adequate social support not only serves as a protective factor for individuals navigating health crises across diverse medical conditions but also correlates with a reduction in medication dependency, expedited recuperation, and enhanced adherence to therapeutic regimens (45). It is imperative to enhance objective support mechanisms and optimize the utilization of social support by patients, thereby maximizing the efficacy of such support systems and mitigating the burden of disease.

### 4.2 Current status of glycemic control in T2DM patients

The investigation revealed that the glycemic control level among patients with T2DM was (8.85 ± 2.107), surpassing that observed in Polish diabetic cohorts as reported by Cyranka et al. (46) (7.11 ± 1.0) and falling below the corresponding level found in Turkish diabetic subjects in the investigation by Celik et al. (23) (9.98 ± 1.80). This discrepancy underscores the discernible variability in the prevailing state of glycemic control among patients across diverse geographic regions. Notwithstanding the intermediary status of glycemic control observed in the subjects of this investigation, it demonstrated a noteworthy elevation compared to the established normative threshold (7.0%) (6). Nonetheless, the incidence of suboptimal glycemic control persisted at a considerable level. Prolonged exposure to elevated blood glucose levels in patients is known to instigate the onset of macrovascular complications, such as cardiovascular diseases (47), microvascular complications, including retinopathy and nephropathy (48), thereby amplifying the overall risk of mortality (49). Hence, it is recommended that clinical practitioners fortify the regimen of glycemic control in diabetic cohorts to ameliorate adverse pathological outcomes, augment the quality of life for patients, and mitigate the economic burdens associated with the condition.

### 4.3 Impact of alexithymia on glycemic control in patients with T2DM

The current investigation elucidated a positive correlation between alexithymia and glycemic control among patients diagnosed with T2DM, aligning with the findings reported by Celik et al. (23). This concordance implies that individuals exhibiting alexithymic traits tend to manifest inferior glycemic control in comparison to their non-alexithymic counterparts. The failure to recognize body symptoms and emotion perceptions could lead to a further incomprehensible psychological and physical suffering, due to poorly regulated diabetes, which may limit the ability to manage their metabolic disease (50). Within the diabetic population, heightened psychological stress may recurrently or persistently activate glucose metabolic pathways, culminating in aberrant glucose concentrations beyond the normative spectrum. Such perturbations in metabolic homeostasis contribute to an inability to sustain glucose levels within physiological bounds, thereby fostering suboptimal glycemic control. Furthermore, psychological stress exerts a deleterious influence on patient self-management, diminishing adherence to therapeutic regimens and consequently engendering compromised glycemic control (51). Alexithymia emerges as a significant psychological determinant contributing to compromised glycemic control (52). Individuals characterized by alexithymic features tend to defer their pursuit of assistance, owing to challenges in articulating and discerning their personal emotional states. This delay, coupled with a reduction in others’ capacity to perceive the patient’s needs accurately, results in a lapse in the timely fulfillment of the patient’s requisites. This circumstance amplifies the psychological burden borne by the patient and diminishes adherence to the prescribed therapeutic interventions, ultimately culminating in suboptimal glycemic control (23). Nevertheless, in the investigations conducted by Mnif and Hintistan et al (15, 53) concerning alexithymia and glycemic control in T2DM patients, the establishment of a conclusive correlation between glycemic control and alexithymia has not been discerned. This absence of a clear association may be attributed to idiosyncrasies within the sampled populations and variances in the methodologies employed for measurement. Consequently, the inquiry into the interrelation between alexithymia and glycemic control necessitates augmentation through additional related studies to enhance the overall persuasiveness of the research.

### 4.4 Impact of social support on glycemic control in patients with T2DM

The study findings indicate an inverse relationship between glycemic control and social support; specifically, a diminished level of social support correlates with a deterioration in glycemic control among diabetic patients. This concurrence aligns with the outcomes reported by Castillo-Hernandez et al. (54). Psychosocial stressors may also lead to decreased immune surveillance as well as abnormal activation of the autonomic nervous system (ANS) and the hypothalamic-pituitary-adrenal axis (HPA), which may affect the patient’s control of the disease (12). In the context of T2DM, social support encompasses emotional, material, and informational facets, serving as a facilitator for enhancing patient adherence to medication protocols, blood glucose monitoring, and lifestyle modifications (e.g., dietary control, physical exercise) (55). This multifaceted support structure aims to ameliorate patients’ self-management proficiency and, consequently, elevate glycemic control, thereby augmenting the overall efficacy of disease management. Conversely, a paucity of social support may engender a deficiency in requisite medical information and assistance for patients, diminishing their cognizance of the ailment. This may result in a procrastination of disease intervention, thereby impinging upon patients’ self-management capabilities and exerting a deleterious impact on glycemic control.

Furthermore, social support may exert a detrimental impact on glycemic control through its association with depression, a phenomenon akin to the observations made by Burns et al. (56) in diabetic patient cohorts. Beyond the direct provision of tangible and spiritual support, the influence of social support on patients’ psychological state emerges as an additional mechanism by which it can affect glycemic control. Social support serves to mitigate psychological stress, assuage adverse emotional states, empower individuals to confront challenges, and enhance self-efficacy in surmounting obstacles (57). Conversely, inadequate social support may precipitate feelings of isolation, helplessness, and anxiety in recipients, potentially culminating in depressive states. Patients enduring chronic depression may experience a decline in confidence regarding their therapeutic regimen, fostering a lack of motivation for self-management and control. This, in turn, can contribute to suboptimal glycemic control.

### 4.5 Effect of depression on glycemic control in T2DM patients

The findings further revealed a positive association between depression and blood glucose control, indicating that elevated depression scores correlate with a deterioration in blood glucose regulation, consistent with the observations of Gonzalez et al. (58). Depression may precipitate physiological alterations in patients, contributing to suboptimal glycemic control in diabetic individuals. Mechanistically, this influence is manifested through the activation of the hypothalamo-pituitary-adrenal axis, stimulation of the sympathetic nervous system, and an escalation in inflammatory responses and platelet aggregation (59). Furthermore, depression may exacerbate the clinical condition and heighten the susceptibility to complications. Secondly, depression can instigate alterations in patients’ attitudes and behaviors towards the ailment, diminishing their inclination to actively engage in treatment and detrimentally impacting self-management facets such as dietary practices, exercise, glucose monitoring, and medication adherence (60, 61). This, in turn, exerts an adverse influence on glycemic control, potentially escalating the severity of the disease, amplifying medical expenditures, and heightening the likelihood of diabetic complications and mortality (62). Moreover, depression may disrupt patients’ social functioning, with chronically depressed individuals experiencing a reduction in social engagement and a decline in overall quality of life. These factors may further compromise the proficiency of glycemic control.

### 4.6 Limitations

This study constitutes a single-center cross-sectional investigation, potentially compromising the representativeness of the encompassed population. Furthermore, the limited sample size may pose a constraint on the generalizability of the findings. To enhance the robustness of future inquiries, multicenter studies incorporating a more diverse diabetic population could be considered, thereby bolstering the external validity of the results. Additionally, intervention studies investigating the impact of alexithymia on glycemic control could be undertaken to augment the persuasiveness of the outcomes.

### 4.7 Conclusions

In this investigation, we formulated a structural equation model encompassing social support, depression, alexithymia, and glycemic control. We scrutinized the intricate pathways through which these factors exert influence on glycemic regulation in diabetic patients. Our findings suggest that enhancing the level of social support and conducting timely assessments of mental health are imperative measures. These interventions aim to ameliorate the physical and psychological stress experienced by patients, subsequently elevating patients’ adherence to treatment and self-management practices. This, in turn, contributes to an enhancement in glycemic control among individuals afflicted with T2DM and those at risk. The optimization of glucose control not only serves to retard the progression of complications and mitigate the risk of mortality but also endeavors to enhance the overall quality of life for patients. Simultaneously, such interventions aspire to alleviate both social and economic burdens associated with T2DM.

## Data Availability

The original contributions presented in the study are included in the article/**Supplementary Material**. Further inquiries can be directed to the corresponding author.
